# Large-Scale Structure-Based Prediction of Stable Peptide Binding to Class I HLAs Using Random Forests

**DOI:** 10.3389/fimmu.2020.01583

**Published:** 2020-07-22

**Authors:** Jayvee R. Abella, Dinler A. Antunes, Cecilia Clementi, Lydia E. Kavraki

**Affiliations:** ^1^Department of Computer Science, Rice University, Houston, TX, United States; ^2^Center for Theoretical Biological Physics, Rice University, Houston, TX, United States; ^3^Department of Chemistry, Rice University, Houston, TX, United States

**Keywords:** structural modeling, random forests, machine learning, HLA-I, peptide binding, docking, immunopeptidomics, antigen presentation

## Abstract

Prediction of stable peptide binding to Class I HLAs is an important component for designing immunotherapies. While the best performing predictors are based on machine learning algorithms trained on peptide-HLA (pHLA) sequences, the use of structure for training predictors deserves further exploration. Given enough pHLA structures, a predictor based on the residue-residue interactions found in these structures has the potential to generalize for alleles with little or no experimental data. We have previously developed APE-Gen, a modeling approach able to produce pHLA structures in a scalable manner. In this work we use APE-Gen to model over 150,000 pHLA structures, the largest dataset of its kind, which were used to train a structure-based pan-allele model. We extract simple, homogenous features based on residue-residue distances between peptide and HLA, and build a random forest model for predicting stable pHLA binding. Our model achieves competitive AUROC values on leave-one-allele-out validation tests using significantly less data when compared to popular sequence-based methods. Additionally, our model offers an interpretation analysis that can reveal how the model composes the features to arrive at any given prediction. This interpretation analysis can be used to check if the model is in line with chemical intuition, and we showcase particular examples. Our work is a significant step toward using structure to achieve generalizable and more interpretable prediction for stable pHLA binding.

## 1. Introduction

Class I Major histocompatibility complexes (MHCs), also known as Human Leukocyte Antigens (HLAs) for humans, are the major players in the endogenous peptide presenting pathway. In this pathway, HLA receptors are loaded with intracellular peptides of length 9–11 amino acids (1). If the binding is stable enough, the resulting peptide-HLA (pHLA) complexes will end up traveling from the endoplasmic reticulum (ER) all the way to the cell surface (2). Surveilling T-cells can inspect the pHLAs and engage an immune response, particularly when the inspected cell is diseased and is presenting immunogenic peptides (i.e., peptides capable of triggering T-cells for being somewhat unusual relative to self peptides). This mechanism is one of the ways personalized immunotherapy has been used to attack tumor cells, through for example, finding T-cells that can target tumor-specific peptides being presented by the patient's HLAs (3).

Inside the ER, peptides are loaded onto the HLA, which is in a peptide-receptive state (4). Thus, peptides that make it to this stage are “trial” bound to the HLA, and only stable complexes make it to the cell surface. Experimentally, mass spectrometry (MS) can be used in combination with acid elution to identify the peptides that are found on the cell surface. Therefore, peptides found with MS can be assumed to be stable (5). However, in the context of personalized immunotherapy, being able to run a fast and accurate computational screening for stable pHLAs would save time and reduce costs, thus prioritizing resources and contributing to better outcomes.

Most of the current methods are based on building a model to predict binders/non-binders using peptide sequences (6–8). NetMHC is the most popular method that trains a neural network to classify binders/non-binders using a dataset of experimental binding affinity measurements (7). It was later shown that binding stability (half-life) is a better measure for immunogenicity over binding affinity, and NetMHCstab was developed using a dataset of experimental half-life measurements (9). While stability may be a more relevant quantity to immunogenicity, more data is available for binding affinity (10), and so NetMHC remained a popular method for binder/non-binder classification. That is until the rise of availability of MS data (11), which can provide a plentiful source of peptide binders directly eluted from different cell types (12, 13). Methods such as NetMHCpan have also used MS data, along with data from experimental binding assays, to enhance the prediction of binding affinity (14). Peptides found with MS can be assumed to have somewhat high binding affinities, since low affinity binders are probably lost during the steps preceding peptide elution. In addition, peptides found with MS can also be assumed to be stable binders, since the pHLA complex must have been stable enough to make it to the cell surface. Hence, methods that use MS data for training are implicitly predicting both affinity and stability. In this work, a large portion of our dataset is derived from MS data for training a predictor of HLA-binders from structure.

Prediction models are typically built on a per-allele basis, so that the only information required for training are peptide sequences known to bind to that specific allele (7, 8). Approaches, such as NetMHC, are then restricted to alleles which have sufficient experimental data. In this work, we are interested in developing a *single* model to classify binders from non-binders across any allele. Such models are also known as pan-allele models and are trained using all of the available pHLA data as one dataset. Thus, pan-allele models have the potential to generalize across alleles and provide accurate predictions for a given pHLA even if little or no experimental data exists for the particular HLA. Sequence-based methods, such as NetMHCstabpan and NetMHCpan, have been developed for this purpose (14, 15). However, pHLA structures could also form the basis for generalizable models, which could work for any allele. If the stability of a pHLA complex is most directly influenced by the chemical interactions found in the structure, then a machine learning algorithm can be used to map these interactions to stability. The information that is specific for a given allele is implicitly encoded in the interactions found in the structure, so any pHLA structure can be treated in the same way during training (i.e., no sequence or structural alignment is required). In other words, machine learning models developed in this formulation are automatically pan-allele.

Another benefit of the model described in this work, which combines the use of structural information with less complex machine learning methods, is greater interpretability. While sequence-based methods such as NetMHCstabpan and NetMHCpan produce highly accurate predictions, these models are not particularly interpretable since they rely on neural networks. Neural networks arrive at a particular output through repeated, non-linear operations, starting from the input features. Thus, it is difficult to analyze the contribution of a particular feature toward any given prediction. However, machine learning models with less complexity, such as random forests, allow more interpretation (16). In turn, the ability to assess the contributions of particular features and mapping these contributions back to the input pHLA structures can be a powerful tool for checking whether the model is in line with chemical intuition.

Although the use of structural information to create generalizable HLA-binding prediction methods has been pursued by many groups in the past, these efforts have been greatly impaired by the computational difficulty in modeling pHLA structures (17). In addition, the massive number of possible combinations involving different HLA alleles and peptide sequences is significantly greater than the number of pHLA crystal structures determined experimentally (e.g., less than 1,000 structures available in the PDB at the time of this writing). Therefore, the development of structure-based binding prediction methods requires large-scale modeling of pHLA complexes. Unfortunately, previously available approaches for generating pHLA structures either do so in a simplistic manner (e.g., peptide threading) (18) or require running for long times per structure, which renders large-scale modeling infeasible (19–22).

Once a 3D structure has been generated for a given pHLA (e.g., through some type of sampling), it is usually passed to a scoring function, which is a sum of energy-related terms aimed at quantifying the binding strength. The weights of these scoring functions can be optimized for pHLAs, (21) or even for specific HLA alleles (23). For instance, structural features based on energy-related terms from the Rosetta scoring function (along with sequence features) were used as input for machine learning, and applied to a training set of 1,000 structures for a single MHC allele (24). Alternatively, simulations of pHLA structures have also produced accurate binding predictions (25). Methods based on molecular dynamics, such as PB/GBSA, have been used to assess binding strength (26). Monte Carlo approaches, such as the one available in the Rosetta package, have also been used to characterize peptide binding profiles for a given allele (27). Unfortunately, simulation approaches are even more computationally expensive than aforementioned modeling methods, also preventing their use in a large scale. Therefore, more research is needed in using a purely machine learning approach to map structures onto binding strength predictions, which will likely be enabled by the availability of large datasets of pHLA structures.

**Figure 1:**
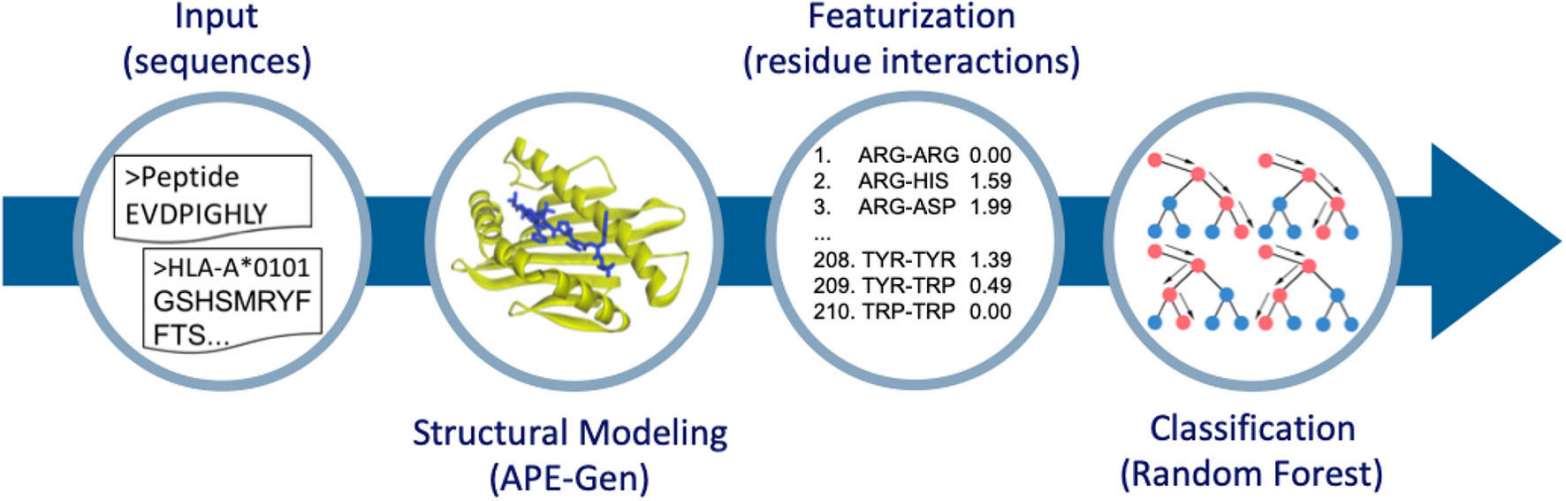
Overview of the method to go from sequence to structure-based features for classification. APE-Gen is used to model pHLA structures, then featurization is done by extracting the residue-residue interactions between peptide and HLA. The final random forest model is trained on these structure-based features.

In this work, we use pHLA structures to predict stable binding. Ideally, a dataset of experimental half-life measurements (like in (15)) that spans multiple alleles would be used here in a regression framework, but such datasets are not readily available or easy to produce. Thus, we rely only on MS data for our source of stable peptide binders, and work within a classification framework (i.e., classifying peptides as stable binders or non-binders). Then, starting from pHLA sequences, we perform large-scale structural modeling. We have recently developed a new method to model pHLA structures called the Anchored Peptide-MHC Ensemble Generator (APE-Gen) (28). APE-Gen has the ability to rapidly generate native-like conformations of pHLA complexes, by leveraging the conserved positioning of the peptide's so called “anchor residues” to particular pockets of the HLA binding cleft. With the development of APE-Gen, we can now use machine learning on pHLA structures on a scale that has not been reached before. The rest of this paper is organized as follows. In the next section, we will describe our approach (Figure 1) of (i) generating pHLA structures, (ii) extracting simple features based on pHLA interactions, and (iii) training a random forest model to classify binders from non-binders. Finally, we show that our model produces high values for the area under the receiver operating characteristic curve (AUROC) in validation tests, and showcase the greater interpretability of the results as compared to neural network approaches. The generated dataset of pHLA structures provides new opportunities to build improved structure-based models to assess pHLA binding, and our model can serve as a benchmark for future models.

## 2. Methods

### 2.1. Generating Peptide-HLA Structures

The dataset of pairs of peptide sequences and HLA alleles was obtained from two different databases. The list of stable binders to a given HLA allele (i.e., positive labels) was taken from a dataset curated by the authors of ForestMHC (16). Their curated dataset is derived from multiple sources, including the SysteMHC Atlas (11), which is a database of eluted peptides from MS experiments. For the polyallelic samples, they used MixMHCpred (29) to deconvolute the samples to a specific allele among a set of multiple well-defined alleles (polyallelic samples). They discarded samples which could come from alleles that MixMHCpred did not support. Note that only 9-mers were considered by ForestMHC (16), so our method was also only trained on 9-mers. However, in principle, other n-mers could be considered by our method as well.

A list of unstable peptides (i.e., negative labels) was obtained from a curated dataset of experimental binding assays, mostly coming from IEDB (30), which was prepared by the authors of MHCFlurry (8). This dataset differs from the previous, as there is an associated value representing the binding affinity measurement of the peptide to the HLA. Since we are interested in finding negative labels, we applied a threshold on the binding affinity with the assumption that low affinity binding implies unstable binding. All pHLA pairs with binding affinity measurements greater than 20,000 nM were extracted from the MHCFlurry dataset. Thresholds are typically set to 500 nM, where peptides with affinity values below this threshold are predicted to be strong enough binders to be presented by the corresponding HLA in the cell surface. Since there do exist peptides that are presented with affinity values greater than 500 nM, we applied a conservative threshold in order TO have more confidence that our dataset of non-binders consists of peptides that are not presented by the corresponding HLA.

Finally, APE-Gen (28) is used to model all of the peptide sequences bound to a given HLA allele. HLAs which did not have a crystal structure available in the PDB were modeled with MODELLER (31), using the corresponding HLA sequence from IMGT (32), and a structural template of an HLA allele from the same supertype classification (33). This is possible due to the conserved tertiary structure of HLA molecules (34), and the fact that alleles within the same supertype share similar peptide binding characteristics (33). Briefly, APE-Gen runs rounds of the following three steps: anchor alignment, backbone reconstruction, and side-chain addition with energy minimization. The list of flexible HLA residues is derived from a list of known important residues for peptide binding (33). A single round of APE-Gen is used per pHLA complex, taking approximately 2 min to model per complex across 6 cores on an Intel Xeon Platinum 8160. The anchor constraint was changed to 4 Angstroms (from the default 2 Angstroms), since it is expected that the anchor interactions of non-binders will be more unstable when undergoing the energy minimization step of APE-Gen. All other parameters are kept at their default (28). For a given pHLA, APE-Gen generates an ensemble of pHLA conformations. This ensemble may include some additional information that is missed when only analyzing a single conformation (35). Therefore, we considered two datasets for training. The first is to simply take the best scoring conformation per pHLA, according to the default scoring function used in APE-Gen, which is Vinardo (36). The second is to take the whole ensemble of each pHLA, pooling every conformation into the dataset. The median number of conformations generated per pHLA is 18. Note that the number of conformations generated per pHLA is not constant due to filtering steps done within APE-Gen (Figure S1). APE-Gen is open-source and available at https://github.com/KavrakiLab/APE-Gen.

### 2.2. Featurization

Each pHLA conformation generated with APE-Gen is then transformed into a feature vector containing information on the residue-residue interactions between the peptide and HLA. The feature vector for a given conformation contains 210 elements, representing the total number of possible pairings between the 20 amino acids, including interactions between two residues of the same type. Each element represents the amount of a particular type of interaction (for example, between alanines and leucines) found in the conformation. The amount of interaction is quantified as the sum of some function of the residue-residue distances, which is defined as the distance in Angstroms between the nearest two heavy atoms computed using the MDTraj Python package (37). Intuitively, such a function should have a high value for low residue-residue distances and monotonically decrease as the residue-residue distance increases in order to represent the amount of interaction. In this work, we consider three functions: the reciprocal, reciprocal squared, and a sigmoid function (Figure S2). The sigmoid function was chosen such that a value of 0.5 occurs at 5 Angstroms. Residue-residue contacts are usually defined within the range of 4.5-5 Angstroms (38).

As an example, assume the function is simply the reciprocal of the residue-residue distance. Furthermore, assume that the fourth element in the feature vector represents interactions between an arginine and aspartic acid. The order of the interactions may be chosen arbitrarily, but is fixed across all pHLA structures. In this scenario, we start by measuring all the distances between arginines in the peptide and aspartic acids in the HLA, as well as between arginines in the HLA and aspartic acids in the peptide. Then, the value for the fourth element in the feature vector is computed as the sum of the reciprocal values of the measured distances. Note that in this implementation peptide-peptide and HLA-HLA interactions were ignored, since interactions between peptide and HLA are expected to have a more direct contribution to stability. With this featurization process, small values in the feature vector represent little interaction for the particular residue-residue pair. Values of exactly zero indicate that the corresponding interaction was not found in the conformation. Conversely, large values represent instances where there was significant residue-residue contact (i.e., low residue-residue distances) found in the conformation. Note that in our construction, only simple, homogenous features based on residue-residue distances between the peptide and HLA are extracted for the model, as opposed to more complex, heterogenous features that were based on energy terms from a scoring function (24).

### 2.3. Model Selection

Models were chosen with five-fold cross validation using the area under the receiver operating characteristic curve (AUROC) as the main metric. The receiver operating characteristic curve plots the true positive rate and false positive rate across different thresholds of the output probability, where a random guess would produce an AUROC of 0.5 and a perfect classifier produces an AUROC of 1.0. We tried three different classifiers, namely logistic regression, gradient boosting, and random forest, across a variety of parameterizations and featurization functions. For each model type, we also tested whether the use of the whole ensemble of conformations improved the final AUROC score. The implementation of the models and analysis is done using Scikit-Learn (39).

## 3. Results

### 3.1. Generalizability

#### 3.1.1. Random Forest Was the Most Robust Model

The final dataset consisted of 155,562 pHLA structures across 99 different alleles, which is to-date the largest dataset of modeled pHLA structures. Within this dataset, 43 alleles have available experimental data on both binders and non-binders. The identity of all modeled pHLAs in this dataset can be found in the Supplementary Material. In total, about 300,000 CPU-hours were required to generate the dataset. There is an approximately 70:30 binders/non-binders ratio across the two sources of data, so class weights were adjusted for all models given the imbalance of class labels. The five-fold cross validation results of the three classifiers tested can be found in Table 1. These results relate to the use of the best parameters found for each type of model, across the three different featurization types. Results for all tested parameters can be found in Tables S1–S3. We find that across the parameters tested, logistic regression performs the worse, while random forest and gradient boosting classifiers give the most robust results as the AUROC values are consistently high with little variation. The overall best performing model was based on random forests (average AUROC: 0.978) with an ensemble of 1,000 decision trees, which use about 7 features (log_2_210) and Gini impurity to determine the quality of a split.

**Table 1:** Average AUROC values from five-fold validation tests across different classifiers and different featurizations.

**Model**	**Feat**	**AUROC**
rf	1/*d*	0.978 (0.000)
rf	1/*d*^2^	0.976 (0.001)
rf	sig	0.975 (0.001)
gb	1/*d*	0.970 (0.002)
gb	1/*d*^2^	0.970 (0.001)
gb	sig	0.977 (0.001)
lr	1/*d*	0.875 (0.003)
lr	1/*d*^2^	0.880 (0.002)
lr	sig	0.882 (0.001)

*Only the best parameters per classifier are shown. rf stands for random forest, gb stands for gradient boosting, and lr stands for logistic regression. Average AUROC values are reported along with standard deviations. Random forest classifiers produce the most robust models*.

#### 3.1.2. Ensemble Dataset With Sigmoid Featurization Improves Performance

With the random forest model, we tested whether training with the whole ensemble of conformations produced by APE-Gen could further increase the AUROC. This dataset consists of 2,825,185 data points with an average of about 18 conformations per pHLA. All of the conformations for a given pHLA are pooled together with the same appropriate label. Across the three different featurization types, a random forest model was trained on the ensemble-enriched dataset of pHLA conformations. Therefore, when testing on an unseen pHLA, APE-Gen is first run to generate an ensemble of conformations. Each featurized conformation is then classified with the model and the output probabilities are averaged to produce the final output. The five-fold cross validation results using the random forest model across the featurization and dataset types are presented in Table 2. We find that across all the different configurations, the best performing random forest model uses the sigmoid-based featurization and the ensemble-enriched dataset with an average AUROC of 0.990. We also found that the ensemble-enriched dataset improves the performance of the other types of models (Table S4) with the gradient boosting model (with sigmoid featurization) also achieving a high average AUROC of 0.982.

**Table 2:** Average AUROC values from five-fold validation tests across different featurizations and different datasets.

**Feat**	**Data**	**AUROC**
1/*d*	Single	0.978 (0.000)
1/*d*^2^	Single	0.976 (0.001)
sig	Single	0.975 (0.001)
1/*d*	Ensemble	0.987 (0.001)
1/*d*^2^	Ensemble	0.988 (0.000)
sig	Ensemble	0.990 (0.000)

*Average AUROC values are reported along with standard deviations. The best model uses sigmoid-based features trained on the ensemble-enriched dataset*.

Sigmoid-based features perform best since higher values are achieved at distances where residue-residue contacts are typically defined (Figure S2). The positive effect of the ensemble may be due to two reasons. First, interactions that are present in multiple conformations for a given pHLA could be an indication for stable interactions, which are now present in the data that is used to train the model. Second, APE-Gen produces on average about 5 more conformations for a true binder than it does for a true non-binder (Figure S1). The additional conformations for binders could turn into bias that the model has learned from.

#### 3.1.3. Final Model Is Competitive With Sequence-Based Approaches on Leave-One-Allele-Out Tests

While our random forest model achieves a high average AUROC on standard five-fold cross validation tests, a tougher test for generalizability would be to partition the train/test split based on the HLA allele. A method that can perform well for tests on unseen allele data would be valuable for cases where pHLA binding prediction is to be done for rarer alleles, with little to no experimental data available. To simulate this scenario, we set aside data related to all the associated examples for a given HLA allele (i.e., both positive and negative examples). We then trained a random forest model using the same procedure described above on the rest of the data. The same procedure for training and testing was then repeated for each one of the HLA alleles in the dataset. This validation setup is called “leave-one-allele-out,” and has been used before in testing NetMHCpan (14). We performed the validation setup across the set of 43 alleles for which there are both positive and negative examples in our dataset, and compared our approach to 5 sequence-based methods: NetMHCstabpan 1.0, NetMHCpan 4.0, NetMHC 4.0, MHCFlurry 1.4.3, and MixMHCpred 2.0.2.

**Figure 2:**
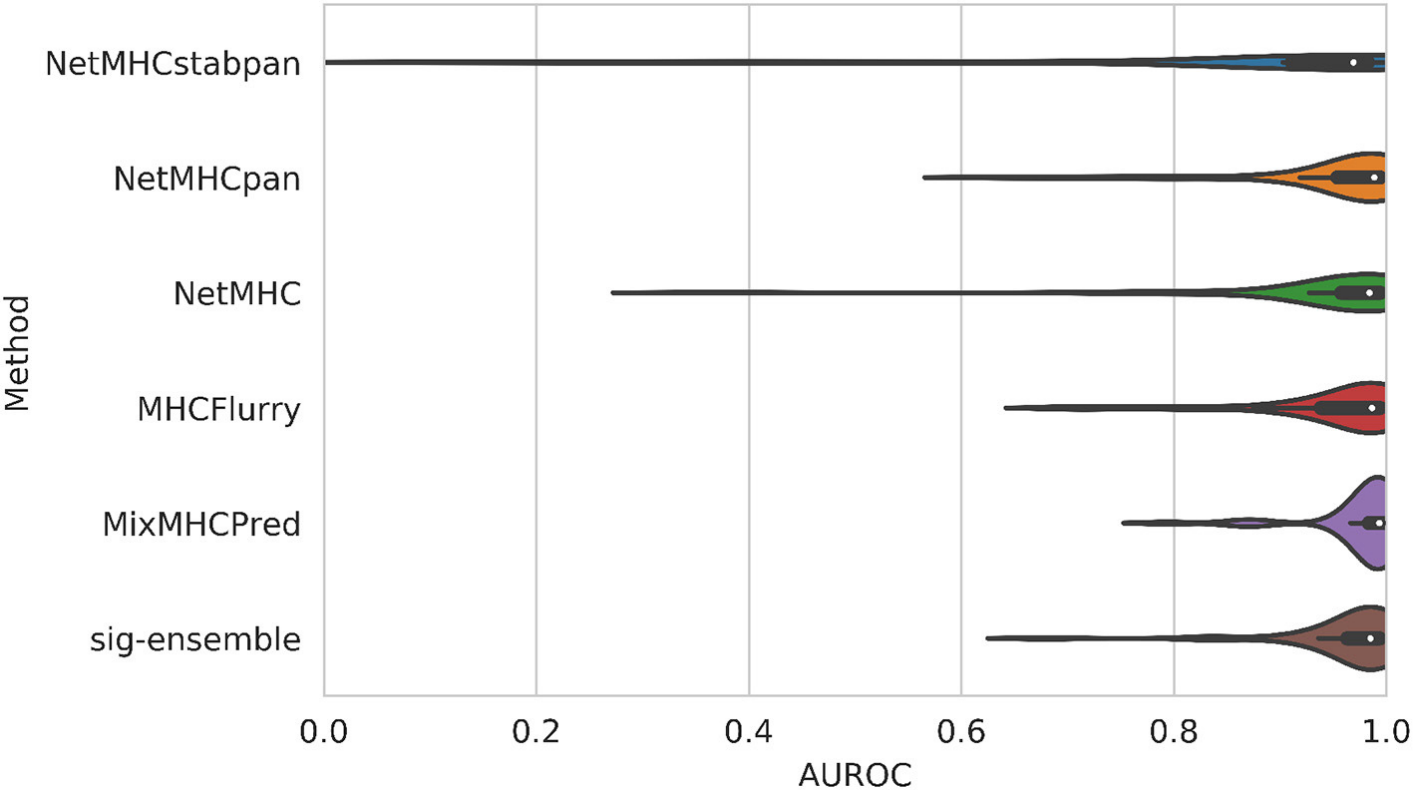
Comparison of AUROC values for leave-one-allele-out experiments. Our structural method based on using sigmoid featurization and ensemble-enriched dataset achieves competitive results with sequence-based methods.

The distribution of AUROC values across all alleles tested can be seen in Figure 2, and corresponding AUROC values can be found in the Supplementary Material. Our method achieves a median AUROC of 0.985 which is greater than NetMHCstabpan (0.969) and competitive with NetMHCpan (0.989). Additionally, the overall distributions of AUROC values shows that our method is more robust (smaller variations) than the other methods, achieving AUROC values greater than 0.9 for all but three alleles (namely HLA-B*39:06, HLA-C*04:01, and HLA-C*14:02). The one exception was MixMHCpred, which achieves the highest median AUROC (0.993) and good robustness. This result is not too surprising since this method was used in the construction of the positive labels (16). Despite having lower median AUROCs against some methods, the difference was never more than 0.01.

We also note that the comparison with NetMHCpan is not particularly fair since there is overlap between the data used to train NetMHCpan and the allele-based validation sets discussed here. In fact, 46% of the data from this work is included in the training set for NetMHCpan. However, this set could not be removed since the overlap is largely on our set of negative labels. Therefore, removing them would complicate the interpretation of AUROC values, and AUROC values cannot even be computed when there are no negative labels.

Other models were also tested in the leave-one-allele-out framework using both the single conformation and ensemble-enriched datasets. The difference in average AUROC between the random forests model (AUROC: 0.985) and the gradient boosting model (AUROC: 0.939) is significant unlike the five-fold cross-validation case. This difference can be qualitatively seen in Figure S3: the distribution shape for the random forests model (ensemble-enriched) reveals the higher concentration of samples closer to 1.0. We speculate that the higher performance of random forests may be due to its robustness in overfitting due to sampling random subsets of data in the training process. We note that our tuning process could be made more exhaustive, and it is conceivable that gradient boosting could perform just as well as random forests for this data. However, it is unclear as to whether gradient boosting could achieve the same interpretability capabilities as random forests.

Finally, we can also compare the performance on a per-allele basis by comparing the AUROC of our method against the others for a given allele. Against NetMHCstabpan, our method improves the AUROC by 0.116 (mean across alleles), while against NetMHCpan, our method improves the AUROC by 0.010. When compared to methods that were trained on a per-allele basis, our method improves the AUROC by 0.018 for NetMHC and 0.010 for MHCFlurry, but is lower against MixMHCpred (0.013). Nevertheless, our method can achieve high AUROCs across alleles in a manner that is competitive against sequence-based methods on average with improved robustness.

### 3.2. Interpretability

The fact that our model is based on random forest also offers a significant interpretability advantage. For instance, we can compute an importance value for each feature of the random forest model by finding the mean decrease in impurity across all the decision trees in the ensemble. We found that most of the top features were hydrophobic interactions with an average importance value of 0.5% over the global average of 0.4%. The least important features include interactions that are less frequent in the dataset, such as ASN-ASN, HIS-HIS, and MET-MET. The full set of feature importance values can be found in the Figure S3.

While computing feature importances provides a global view of what the model is learning, we can also inspect how the model arrives at a prediction for a particular example (40). The prediction output of the random forest model, *P*(*x*), for a particular example *x* is a probability to be in the positive class (which is thresholded by 0.5 to classify as stable binder/non-binder). The output can be decomposed as

(1) $$\begin{array}{l} P\left(x\right)=\text{bias}+\overset{210}{\underset{j=1}{\sum }}{\text{contri}}_{j}\left(x\right) \end{array}$$

where the bias term reflects the ratio of positive examples in the data, which is 0.5 in this work because the classes were reweighted from the model. The interesting quantity is contri_*j*_(*x*), which is the contribution of feature *j* toward the prediction output. This equation tells us that the contributions are then combined in a linear manner reflecting how the decision trees split on a single feature at a time. The contribution values can be positive (contribute toward the stable binding) or negative (contribute toward unstable binding). Furthermore, since each residue-residue interaction was added to the corresponding element in the feature vector in a linear manner, we can decompose the contribution values further across every possible residue-residue contact in the original pHLA structure. While the contribution values are computed in a non-linear way based on the values of the other features across the training dataset, we can still inspect the features that greatly contribute to the prediction for a given example and test if they are in line with chemical intuition. The feature contributions are computed with the Python package treeinterpreter (40).

As an example, we model the structure of the peptide, EVDPIGHLY, bound to HLA-A*01:01. This is a peptide that has been used as a target for T-cell-based immunotherapy against melanoma (41). Our model correctly predicts that this peptide is a stable binder, so we analyze the feature contributions leading to this prediction. The anchor residues for this allele are in position 2 (VAL) and position 9 (TYR), and we find that anchor-related interactions account for 26% of the positive contributions. However, our model is finding a significant positive contribution from other interactions. The feature with the largest position contribution is the ASP-ARG interaction (13%). In fact, position 3 is an aspartic acid (ASP), and interactions involving position 3 have the largest total positive contribution (32%). Interestingly, it is known that aspartic acid is a “preferred” residue in position 3 for peptides binding to HLA-A*01:01 (30).

When we model a destructive mutation on the anchor residue in position 2, from VAL to TRP, our model predicts that the new peptide is unstable. As expected, the feature contributions indicate that 42% of the negative contributions come from the TRP in position 2. Thus, the model is indeed using the interactions introduced by this mutation.

Our models are publicly available, alongside the ability to do the interpretation analysis presented in this section. The interpretation analysis has been automated to be able to produce summarized results as well as the raw data. This data contains more information than presented in this section, including a decomposition of the contribution values across each peptide-HLA residue-residue pair. The structural modeling with APE-Gen, classification with random forest, and interpretation analysis can be done for any pHLA of interest, and is available as an easy to use Docker image (https://hub.docker.com/r/kavrakilab/apegen/tags) with a tutorial found in https://github.com/KavrakiLab/pHLA-RFclassifier-from-structure.

## 4. Discussion

In this work, we performed large-scale modeling of pHLA conformations, which is used to train an interpretable, structure-based classifier for pHLA binding prediction. With APE-Gen as the enabling technology, we generated a dataset of pHLA conformations that is the largest of its kind, opening the door for machine learning to be performed on top of pHLA conformations. We investigated various featurizations that are solely based on simple, homogenous conformational features (i.e., peptide-HLA, residue-residue distances). We show on our dataset that our model achieves competitive AUROCs against sequence-based methods. Additionally, our model based on random forest offers an interpretability advantage over approaches based on neural networks.

Note that while our dataset of structures is large with respect to structural modeling efforts (e.g., over 150,000 different pHLAs), this number becomes small when compared to the number of sequences that sequence-based methods have been trained on (e.g., about 3 million for NetMHCpan). Additionally, this work has only been tested on 9-mer ligands, but other n-mers do of course exist as binders to a significant extent (42). It should also be noted that our source of positive labels was dependent on the accuracy of MixMHCpred. In order to push the accuracy of our classifier, we need to include all of the available high-quality experimental data for training, which should increase our confidence in the final model. Our classifier does not have any inherent limitation on the peptide length, as APE-Gen can model other n-mers, and the featurization process is also not specific to 9-mers. Future work can focus on modeling more pHLAs, including the proper modeling of longer peptides by APE-Gen.

Despite the efficiency of APE-Gen, the step of modeling a new structure still takes a few minutes. Modeling structures takes significant computational resources, and reaching the scale of training data that sequence-based methods train on requires at least an order of magnitude more computational time. This makes our structure-based classifier slower than a sequence-based method for unseen pHLAs, and currently requires high performance computational resources to make large peptide screenings viable. However, the modeling of pHLA structures would only have to be performed once. Thus in the future, we can try to alleviate this burden by creating a database of previously modeled pHLAs, so that the classifier can skip the modeling step for all previously modeled complexes.

The use of structure may be the reason that we achieve high AUROCs on our dataset despite the relatively small dataset size. Models based on sequence are supposed to infer structural information, like the interactions between peptide and HLA, in order to get to accurate binding predictions. Our construction feeds this information directly to the model, which may be the key for generalizability. In fact, we can confirm when the model is properly using interaction information because our model was based on random forests. Our model can be made transparent, and we can understand *why* the model reached any given prediction. For “black-box” methods like neural networks, the best that can be done would be to try identifying patterns among the highest scoring samples. A list of random peptides could be run through the neural network for a given allele, and then the top scoring peptides can be analyzed for any noticeable peptide binding motif. For any given peptide, one might guess how the neural network arrived to the prediction by reasoning back to the peptide binding motif. This route is indirect at best, since it is extremely difficult to interrogate the neural network into revealing what leads to a particular prediction, which is an inherent problem of this methodology. There is no way of knowing that the reason a peptide was classified as a non-binder was because the model learned to penalize when a TRP exists in the peptide sequence along with a TYR in the HLA sequence, for example; a potentially spurious association with no obvious biochemical reason for affecting the binding. Our random forest model does reveal such information on a *per prediction basis*, as demonstrated in the Results section. For any given prediction, correct or not, we can see how the model composes the features into its final output, and check if it is in line with chemical intuition. This can even be useful for suggesting the kind of additional data needed for training when analyzing an example that was incorrectly classified.

We would like to make it clear however, that the goal of this work is not to produce a method for pHLA binding prediction that will replace the gold-standard methods, such as NetMHC and NetMHCpan, which are available as a public webservers for rapid prediction. Many challenges remain as mentioned in this section. The contribution of this work reveals that for the pHLA binding prediction task, structure-based methods can work as a proof-of-concept. The time investment spent in doing the structural modeling enables the benefit of added interpretability. The residue-residue interactions present between peptide and HLA can be directly extracted as simple features for the model. Additionally, the random forests model can highlight how the features are composed to form the output of any given pHLA. When combined together, one instantly has a link to relate the binding prediction back to each individual peptide-HLA residue-residue interaction for further analysis. Such a capability can be valuable as a complement to sequence-based approaches. For instance, it could be used after epitope discovery efforts, providing more detailed analysis of binding for peptides that are strong candidates as targets for vaccine development, T-cell-based immunotherapy, or as the potential triggers for autoimmune reactions. The obtained structural information could be used to lead peptide optimization efforts, or to provide a molecular basis for the presentation of unusual HLA-binders. As we continue to push the accuracy of our method, our results and dataset of pHLA structures can be used as a benchmark for a new generation of structure-based methods for HLA binding prediction and epitope discovery.

## Data Availability Statement

The dataset of structural features, based on sigmoid featurization, can be found inside a Docker image (https://hub.docker.com/r/kavrakilab/apegen/tags) with a tutorial found in https://github.com/KavrakiLab/pHLA-RFclassifier-from-structure. The dataset of pHLA structures and the other featurized datasets are available upon request.

## Author Contributions

JA conceived the project idea, generated the data, and developed the model. JA, DA, CC, and LK designed the experiments, analyzed the results, wrote, and edited the paper.

## Conflict of Interest

The authors declare that the research was conducted in the absence of any commercial or financial relationships that could be construed as a potential conflict of interest.
