# Supplementary material for: Large-Scale Structure-Based Prediction of Stable Peptide Binding to Class I HLAs Using Random Forests
Source: Front Immunol. 2020 Jul 22;11:1583. doi: 10.3389/fimmu.2020.01583 (PMC7387700; doi:10.3389/fimmu.2020.01583)
Supplement: Supplementary file 1 [file Data_Sheet_1.PDF]

Supplementary Material

1 SUPPLEMENTARY TABLES AND FIGURES

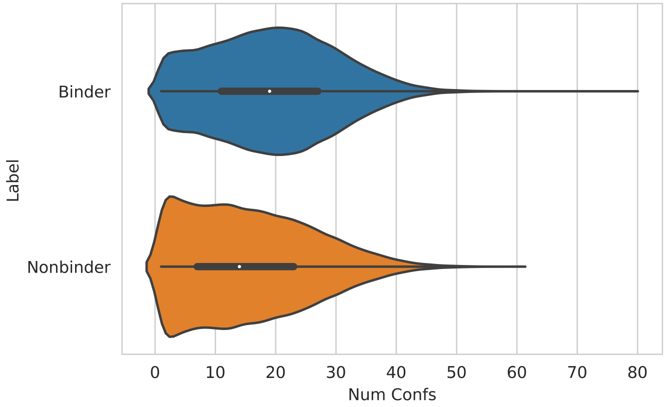

Figure S1: The distribution of the ensemble size per pHLA outputted by APE-Gen, separated by binders and nonbinders. The median number of conformations across the whole ensemble-enriched dataset is 18. The median number of conformations within binders is 19, while nonbinders have a median of 14 conformations.

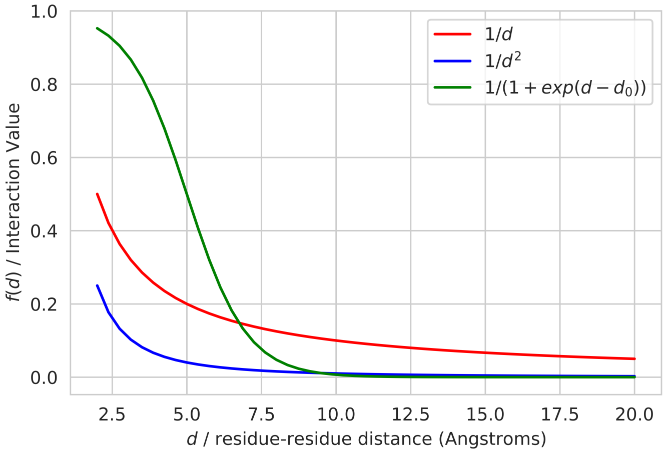

Figure S2: Plot of interaction values as a function of the residue-residue distance. Three different featurization types are shown, which turn small residue-residue distances into large interaction values (and vice-versa). The value of $d_0 = 5$ Angstroms is used for the sigmoid function, which attains a value of 0.5 at a distance of 5 Angstroms and is a typical definition for a contact.

Table S1. Full cross-validation results across all the parameters used for the random forest model. Different featurization functions, numbers of trees, and number of features for splitting were tested. Average AUROC values are reported along with standard deviations.

Feat	Num Trees	Num Feat	AUROC
$1/d$	100	log2	0.975 (0.000)
$1/d$	100	sqrt	0.975 (0.000)
$1/d$	100	0.1	0.974 (0.001)
$1/d$	500	log2	0.978 (0.000)
$1/d$	500	sqrt	0.977 (0.001)
$1/d$	500	0.1	0.977 (0.000)
$1/d$	1000	log2	0.978 (0.000)
$1/d$	1000	sqrt	0.977 (0.001)
$1/d$	1000	0.1	0.977 (0.001)
$1/d^2$	100	log2	0.972 (0.001)
$1/d^2$	100	sqrt	0.972 (0.001)
$1/d^2$	100	0.1	0.971 (0.000)
$1/d^2$	500	log2	0.976 (0.001)
$1/d^2$	500	sqrt	0.975 (0.001)
$1/d^2$	500	0.1	0.974 (0.001)
$1/d^2$	1000	log2	0.976 (0.001)
$1/d^2$	1000	sqrt	0.975 (0.001)
$1/d^2$	1000	0.1	0.974 (0.001)
sig	100	log2	0.970 (0.001)
sig	100	sqrt	0.970 (0.001)
sig	100	0.1	0.969 (0.001)
sig	500	log2	0.975 (0.001)
sig	500	sqrt	0.974 (0.001)
sig	500	0.1	0.973 (0.001)
sig	1000	log2	0.975 (0.001)
sig	1000	sqrt	0.974 (0.001)
sig	1000	0.1	0.973 (0.001)

Table S2. Full cross-validation results across all the parameters used for the gradient boosting model. Different featurization functions, numbers of trees, and learning rate values were tested. Average AUROC values are reported along with standard deviations.

Feat	Num Trees	Learning Rate	AUROC
$1/d$	100	0.01	0.724 (0.004)
$1/d$	100	0.1	0.846 (0.003)
$1/d$	100	0.2	0.888 (0.002)
$1/d$	500	0.01	0.800 (0.003)
$1/d$	500	0.1	0.934 (0.002)
$1/d$	500	0.2	0.955 (0.002)
$1/d$	1000	0.01	0.845 (0.003)
$1/d$	1000	0.1	0.957 (0.002)
$1/d$	1000	0.2	0.970 (0.002)
$1/d^2$	100	0.01	0.742 (0.002)
$1/d^2$	100	0.1	0.871 (0.002)
$1/d^2$	100	0.2	0.903 (0.002)
$1/d^2$	500	0.01	0.837 (0.003)
$1/d^2$	500	0.1	0.939 (0.002)
$1/d^2$	500	0.2	0.957 (0.002)
$1/d^2$	1000	0.01	0.872 (0.002)
$1/d^2$	1000	0.1	0.958 (0.002)
$1/d^2$	1000	0.2	0.970 (0.001)
sig	100	0.01	0.761 (0.003)
sig	100	0.1	0.896 (0.002)
sig	100	0.2	0.925 (0.001)
sig	500	0.01	0.862 (0.001)
sig	500	0.1	0.954 (0.001)
sig	500	0.2	0.967 (0.001)
sig	1000	0.01	0.896 (0.001)
sig	1000	0.1	0.968 (0.001)
sig	1000	0.2	0.977 (0.001)

Table S3. Full cross-validation results across all the parameters used for the logistic regression model. Different featurization functions and values of regularization were tested. Average AUROC values are reported along with standard deviations.

Feat	Reg	AUROC
$1/d$	0.1	0.875 (0.003)
$1/d$	1.0	0.875 (0.003)
$1/d$	10.0	0.875 (0.004)
$1/d^2$	0.1	0.870 (0.002)
$1/d^2$	1.0	0.880 (0.002)
$1/d^2$	10.0	0.882 (0.002)
sig	0.1	0.882 (0.001)
sig	1.0	0.882 (0.001)
sig	10.0	0.882 (0.001)

Table S4. Cross-validation results across each model (using sigmoid featurization and the best parameters found for each) tested on the single conformation and the ensemble-enriched datasets. Average AUROC values are reported along with standard deviations.

Model	Data	AUROC
rf	single	0.975 (0.001)
lr	single	0.882 (0.001)
xg	single	0.977 (0.001)
rf	ensemble	0.990 (0.000)
lr	ensemble	0.895 (0.001)
xg	ensemble	0.982 (0.001)

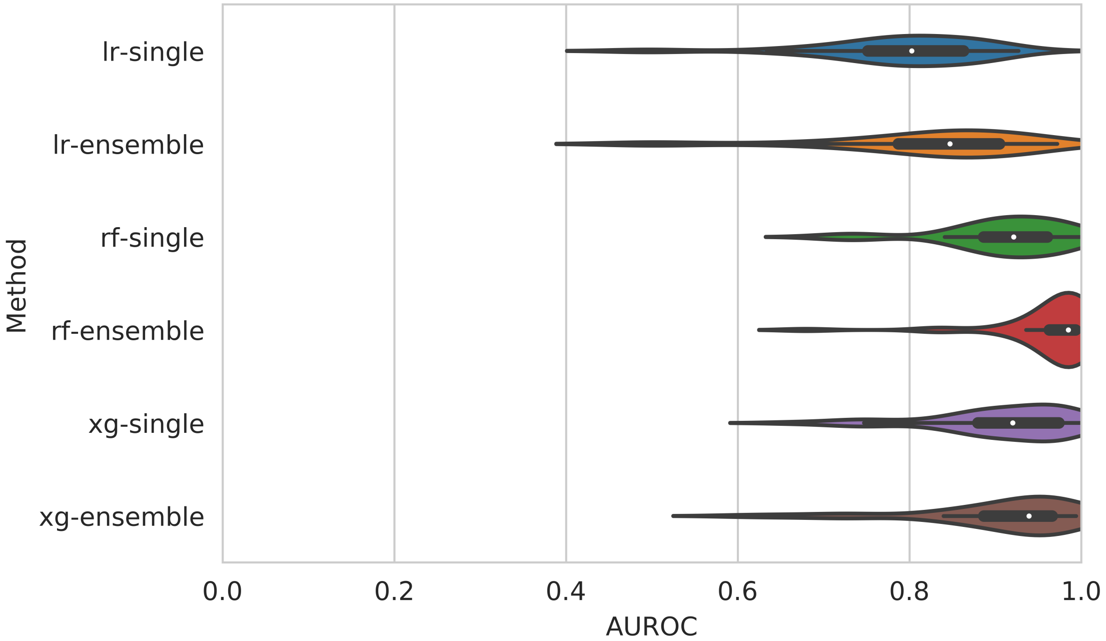

Figure S3: Comparison of AUROC values for leave-one-allele-out experiments across different configurations of our structural method. Different models were considered (using sigmoid featurization and tbe best parameters found for each) and tested on the single conformation and the ensemble-enriched datasets.

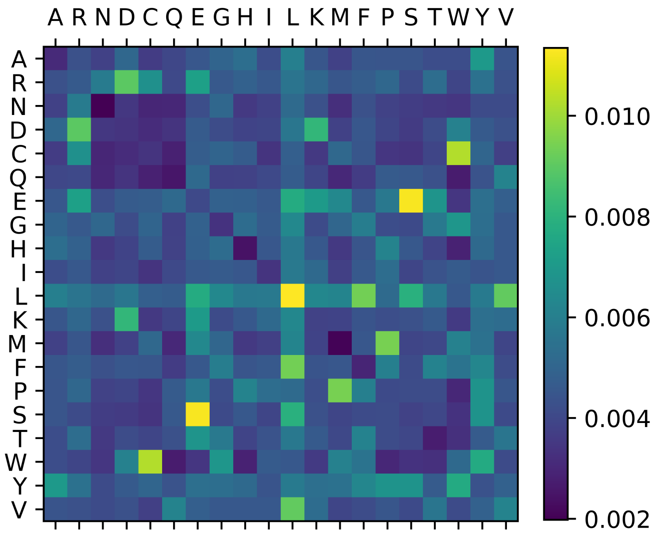

Figure S4: Feature importance values for the final random forest model. Note that the matrix is symmetric across the matrix diagonal. The model relies more on features with higher importance values. Hydrophobic interactions generally have greater importance.
